# A Facile and Eco-Friendly Hydrothermal Synthesis of High Tetragonal Barium Titanate with Uniform and Controllable Particle Size

**DOI:** 10.3390/ma16114191

**Published:** 2023-06-05

**Authors:** Tingting Wang, Xiaoxiao Pang, Bin Liu, Jie Liu, Jing Shen, Cheng Zhong

**Affiliations:** 1 Key Laboratory of Advanced Ceramics and Machining Technology (Ministry of Education), School of Materials Science and Engineering, Tianjin University, Tianjin 300072, China; wtt@tju.edu.cn (T.W.); xiaoxiao_pang17@163.com (X.P.); arthurlou@tju.edu.cn (B.L.); jieliu0109@tju.edu.cn (J.L.); 2 Chongqing Newcent New Materials Co., Ltd., Chongqing 401147, China; shenjing@chinanewcent.com; 3 Joint School of National University of Singapore and Tianjin University, International Campus of Tianjin University, Binhai New City, Fuzhou 350207, China

**Keywords:** barium titanate, high tetragonality, particle size, hydrothermal method

## Abstract

The preparation of tetragonal barium titanate (BT) powders with uniform and suitable particle sizes is a significant prerequisite for ultra-thin and highly integrated multilayer ceramic capacitors (MLCCs). However, the balance of high tetragonality and controllable particle size remains a challenge, which limits the practical application of BT powders. Herein, the effects of different proportions of hydrothermal medium composition on the hydroxylation process are explored to obtain high tetragonality. The high tetragonality of BT powders under the optimal solvent condition of water:ethanol:ammonia solution of 2:2:1 is around 1.009 and increases with the particle size. Meanwhile, the good uniformity and dispersion of BT powders with particle sizes of 160, 190, 220, and 250 nm benefit from the inhibition of ethanol on the interfacial activity of BT particles (BTPs). The core–shell structure of BTPs is revealed by different lattice fringe spacings of the core and edge and the crystal structure by reconstructed atomic arrangement, which reasonably explains the trend between tetragonality and average particle size. These findings are instructive for the related research on the hydrothermal process of BT powders.

## 1. Introduction

The excellent dielectric and ferroelectric properties of barium titanate (BT) make it stand out in the application of dielectric layers for multilayer ceramic capacitors (MLCCs) [1,2]. Due to the development of ultra-thin devices and the requirements of high integration, tetragonal BT powders with appropriate and uniform particle sizes are preferred [3,4,5]. Generally, the traditional synthesis of BT powders mostly adopts the solid-state method, that is, the reaction of BaCO_3_ and TiO_2_ at a high temperature above 1100 °C [6,7]. However, the high-temperature preparation process is prone to agglomeration and difficult to control the particle size, which adversely affects the properties of the subsequent sintered body [2,8]. As a typical liquid phase method, the sol-gel method is difficult to achieve mass production [9]. The high concentration of surfactants in the microemulsion method restricts the development from the perspective of cost and environmental protection [10]. In contrast, the hydrothermal method makes it possible to prepare BT particles (BTPs) under milder conditions and is of particular interest due to its energy saving, facile operation, and eco-friendly feature [11]. Most importantly, a simple and eco-friendly synthesis strategy avoids the use of toxic surfactants and the introduction of surface impurities in particles [12].

The degree of tetragonal distortion of BT (tetragonality, the ratio of the lattice parameter *c* to *a*) caused by the eccentric displacement of titanium atoms is commonly used to measure the dielectric properties [13,14]. The higher the ratio of *c/a*, the higher the dielectric constant of the BT powder [15]. Typically, a high *c/a* ratio and suitable particle size are essential to obtain high-performance BT powders. The crystal structure and dielectric properties of BTPs are also affected by their shape, size, and uniformity [16]. Nevertheless, previous studies have shown that the structure of BT powders obtained by traditional hydrothermal methods generally presents the cubic phase or a small amount of tetragonal phase, which is unfavorable for the dielectric properties [17,18]. Subsequent high-temperature heat treatment is often needed to improve its tetragonality [2]. Furthermore, most efforts have focused on the size effect on the phase transformations of BT [19], while the controllable particle size and high tetragonality are still difficult to balance. Recently, Nakashima et al. [20] reported that when TiO_2_ and Ba(OH)_2_·8H_2_O were used as raw materials, the hydrothermal temperature could adjust the particle size of BT powders to a certain extent, but the tetragonality and morphology of obtained nanocubes were not satisfactory, which was not conducive to obtaining high dielectric properties.

In addition to the adjustment of hydrothermal parameters, the strategy of introducing additives to adjust the particle size and tetragonal has also been tried. Peng et al. [21] showed that the average particle sizes of BT powders were only changed from 71.86 nm to 77.34 nm by regulating the concentration of polyethylene glycol. Additionally, excessive concentration of polyethylene glycol instead generated undesired cubic phases and reduced the tetragonality [21]. Moreover, the uniform morphology of BT powders is also crucial for the reliability of MLCCs. Interestingly, the morphology of BTPs is related to the reaction medium. Maček Kržmanc et al. [22] found that BTPs with cubic morphology were synthesized by adding ethanol into the hydrothermal medium. Habib et al. [23] synthesized tetragonal BT powders using TiO_2_ and Ba(OH)_2_·8H_2_O as precursors in a mixed solvent of equal proportions of ethanol and water for several days but only obtained the tetragonality of about 1.007 and the particle sizes ranging from 90 nm to 120 nm. Thus, the introduction of ethanol and its proportion in the hydrothermal solvent are worth further discussion. Given the above, the preparation of BT powders with high tetragonality, controllable particle size, and uniform morphology is still a significant and challenging task.

In this work, we prepare four BT powders featured with high tetragonality, good homogeneity, and controllable particle sizes by a facile and eco-friendly hydrothermal method (one-step hydrothermal process without additional heat treatment and additives). The high tetragonality of around 1.009 is obtained with the proposed optimal hydrothermal solvent, and the reason for the high tetragonality is analyzed. Then, BT samples with high tetragonality and controllable particle size are obtained only by simple control. Subsequently, the trend of average particle size and tetragonality and its cause are discussed. The results show that the dual goals of high tetragonality and controllable particle size are achieved while maintaining uniformity and dispersion, which is conducive to further research on expanding the practical applications of BT powders.

## 2. Materials and Methods

### 2.1. Materials

The original materials and purchase information used in this experiment are as follows: TiO_2_ (99%, Meryer Chemical Technology Co., Ltd., Shanghai, China), Ba(OH)_2_·8H_2_O (98%, Shanghai Aladdin Biochemical Technology Co., Ltd., Shanghai, China), ethanol (≥99.7%, Tianjin Yuanli Chemical Co., Ltd., Tianjin, China), ammonia solution (25–28%, Shanghai Aladdin Biochemical Technology Co., Ltd.), acetic acid (30%, Shanghai Aladdin Biochemical Technology Co., Ltd.), and deionized water for laboratory use. All chemicals were used directly without any pretreatment.

### 2.2. Synthesis of BT Powders

Based on our previous research [24], we further explored the optimal solvent ratio and hydrothermal scheme to prepare high tetragonal BT powder with controllable particle size. First, 16.900 g of Ba(OH)_2_·8H_2_O was poured into deionized water and heated in a water bath at 80 °C until dissolved. Then, 1.712 g of TiO_2_ was poured into the well-stirred Ba(OH)_2_ solution, followed by adding ethanol and ammonia solution in turn and stirring without heating. The optimal ratio of water: ethanol: ammonia solution in the hydrothermal medium was 2:2:1. The pH value of the mixed solution reached 13. Then, the mixture was poured into a 50 mL para-polyphenylene (PPL) autoclave and reacted at 260 °C for 20–50 h without a surfactant/stabilizing agent. The BT powders obtained with an interval of 10 h were recorded as BT–160, BT–190, BT–220, and BT–250, which corresponds to the particle size of ~160, ~190, ~220, and ~250 nm, respectively. After the reaction was completed and cooled naturally, the contents of the autoclaves were taken out and centrifuged (10,000 rpm for 5 min) once directly. The products were washed with deionized water and acetic acid solution several times, dried at 80 °C for 12 h, and ground to obtain BT powders.

### 2.3. Materials Characterization

The phase and crystalline structure of four BT powders were determined by X-ray diffraction (XRD, D8 Advanced, Bruker Corp, Bremen, Germany) under Cu Kα_1_ radiation (1.5406 Å) at 40 kV and 40 mA, with 2*θ* angle ranging from 10–90°. The local crystal structures of four BT powders were characterized by Raman spectroscopy with a 532 nm laser (Raman, Horiba LabRAM HR Evolution, Kyoto, Japan) in the Raman shift range of 100–1500 cm^−1^. The uniformity, dispersion, and energy dispersive spectrometer (EDS) quantitative results of four BT powders were evaluated by a scanning electron microscope (SEM, Hitachi S4800, Tokyo, Japan). The particle sizes of 100 particles in each SEM image were measured to calculate the corresponding average particle size. TEM, high-resolution transmission electron microscopy (HRTEM), selected area electron diffraction (SAED), and elemental mapping results were obtained by transmission electron microscopy (TEM, JEOL 2100F, Tokyo, Japan) to study the microstructures and element information of four BT powders.

## 3. Results and Discussion

**Figure 1:**
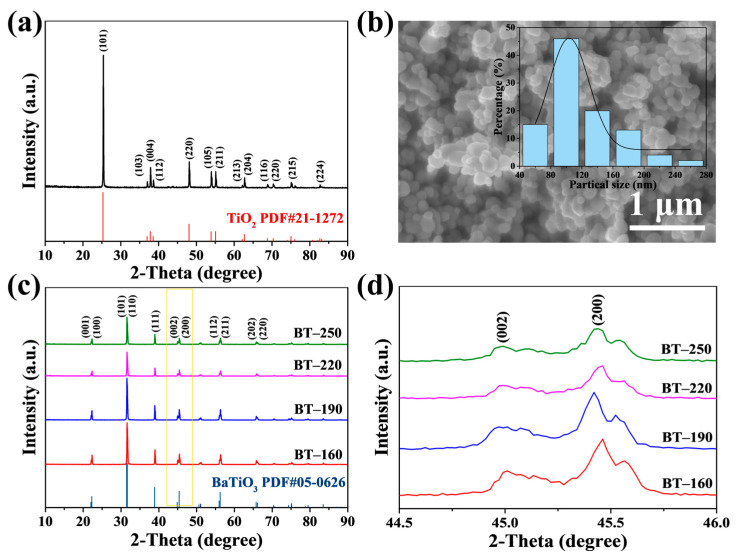
(**a**) XRD pattern and (**b**) SEM image of titanium source. The illustration shows the size distribution histogram of the titanium source; (**c**) XRD patterns (2*θ* = 10–90°); and (**d**) partially enlarged XRD patterns (2*θ* = 44.5–46.0°) of four BT powders with different particle sizes.

BT powders are synthesized by a facile and eco-friendly hydrothermal method using anatase TiO_2_ as the titanium source, which can be seen from the XRD pattern in Figure 1a. This is because anatase TiO_2_ powders have good reactivity with Ba(OH)_2_ solution, and the reaction to generate BTPs is relatively thorough under mild hydrothermal conditions [25]. In this work, BTPs with good uniformity are synthesized by using the agglomerated and uneven TiO_2_ particles (40–280 nm, as shown in Figure 1b). Figure 1c,d show the XRD patterns (2*θ* = 10–90°) and partially enlarged XRD patterns (2*θ* = 44.5–46.0°) of four BT powders with different particle sizes. In Figure 1c, all detectable diffraction peaks indicate that four BT samples with different sizes correspond to tetragonal BT with a space group of *P4mm* (PDF#05-0626). In addition, it has been reported that BT samples with micrometer size demonstrate the same crystal phase (PDF#05-0626) [26]. The strong and sharp diffraction peaks suggest that four BT samples with different particle sizes all have good crystallinity [27]. Moreover, there are no impure peaks (such as BaCO_3_) in the XRD patterns, indicating that the hydrothermal reaction is thorough. In XRD patterns, the tetragonal BT has an obvious splitting peak at 2*θ* = 45°, indicating the asymmetric elongation of the crystal structure along the *c*-axis, while the cubic BT has no splitting peak at 2*θ* = 45° [28]. Therefore, the splitting peaks around 2*θ* = 45° indicate the tetragonal phase structure, corresponding to the (002) and (200) crystal planes, respectively [29]. In order to observe the tetragonal characteristics of BT powders more intuitively, Figure 1d shows the different degrees of diffraction peak splitting. It can be seen that all BT samples split into obvious double peaks at 2*θ* = 45°. This result strongly demonstrates that we have successfully synthesized BT powders with a tetragonal phase by a facile hydrothermal method. Furthermore, the degree of splitting tends to increase with particle sizes; that is, the difference between the diffraction angle 2*θ* values corresponding to the (200) and (002) splitting peaks (Δ2*θ*) increases. This means that the tetragonal characteristics become more and more evident as the particle size increases.

However, it has been proved that BT powders synthesized by hydrothermal methods usually contain tetragonal and cubic phases, which results in the tetragonal properties of BT powders usually being lower than the theoretical value of 1.011 [30]. In general, the existence of lattice defects will lead to lattice vibration, which is essentially different from that of an ideal crystal. During the hydrothermal process, hydroxyl ions are generated, which tend to replace the lattice oxygen of the BT lattice [31]. The formed hydroxyl defects will induce the formation of a cubic phase in tetragonal crystallites [32]. Therefore, the synthesized BT by hydrothermal method usually contains tetragonal and cubic phases. In order to obtain BT powders with high tetragonality, ethanol is introduced into the hydrothermal medium to adjust the phase structure of the products. Experiments with different proportions of hydrothermal solvents show that water: ethanol: ammonia solution = 2:2:1 in the hydrothermal medium is favorable for high tetragonality (Figure 1c and Figure S1–S5). This is because when the content of ethanol is low, the barium ion dissolves in water, and the hydroxyl groups are adsorbed on the titanium sites to aggravate the degree of hydroxylation [32]. However, when the content of ethanol is high, the catalytic oxidation reaction of ethanol occurs at the titanium site, and the adsorption on the lattice oxygen of BTPs leads to severe hydroxylation and BaCO_3_ impurities [32]. More cubic phases in BTPs tend to be associated with the hydroxylation process [32]. Therefore, only when the ethanol in the hydrothermal medium presents in an appropriate ratio is the hydroxylation process suppressed due to reaction competition. Specifically, the tetragonality of BT samples is usually measured by the ratio of the lattice parameters of the *c*-axis and *a*-axis after XRD refinement. According to the calculation of XRD refinement results, the *c*-axis lattice parameter values, *a*-axis lattice parameter values, and tetragonality of four BT powders with different particle sizes are shown in Table S1. All BT samples obtained from Table S1 have high tetragonality, and the tetragonality is indeed consistent with the splitting degree trend of the splitting peak. Furthermore, the ratios of tetragonal and cubic phases in four BT powders are presented in Table S2, and it is clear that the trend of the tetragonal phase ratio is consistent with tetragonality.

Different from the overall analysis of crystal structure by XRD, the sensitivity of Raman spectroscopy to structural symmetry was used to analyze the local structure of BTPs. The lattice vibration modes of cubic phase BT with the structure of the *Pm3m* space group are transformed into 3*F*_1u_ + 1*F*_2u_ according to the irreducible representation [33]. Since both *F*_1u_ and *F*_2u_ modes are Raman inactive, the cubic BT does not exhibit Raman activity [34]. However, the Raman inactive *F*_1u_ and *F*_2u_ modes are split into Raman active *A*_1_, *B*_1_, and *E* vibrational modes in tetragonal BT [33]. The interaction between the dipoles formed by the deviation of Ti atoms from the center of the oxygen octahedron results in the long-range Coulomb force [35]. Under the combined action of crystal symmetry breaking and long-range Coulomb force, both *A*_1_ and *E* modes are split into transverse optical (TO) mode and longitudinal optical (LO) mode [33].

**Figure 2:**
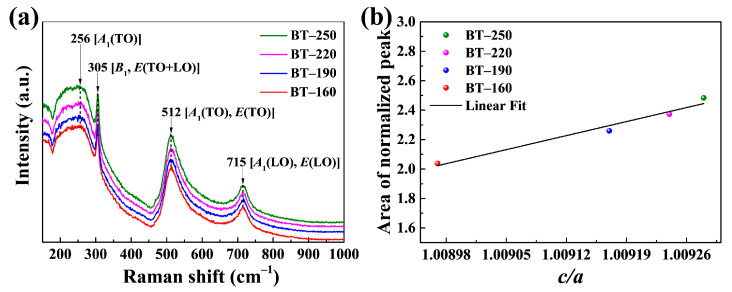
(**a**) Raman spectra of four BT samples with different particle sizes in the 150–1000 cm^−1^ band; (**b**) The relation curve between the area of the 305 cm^−1^ normalized peak and *c/a*.

The Raman spectrum in the 150–1000 cm^−1^ band is the focus of observation. In Figure 2a, the characteristic bands of tetragonal BT are displayed at 256 cm^−1^, 305 cm^−1^, 512 cm^−1^, and 715 cm^−1^. The most characteristic band among them is 305 cm^−1^, which is due to the asymmetry induced by off-center Ti atoms of [TiO_6_] manifesting as the [*B*_1_, *E*(TO + LO)] phonon mode, suggesting the existence of tetragonal distortion inside BTPs [36,37]. The peaks of other bands are related to the propagation and coupling of their corresponding phonon modes in the local structure of tetragonal BT. The broad peaks at the 256 cm^−1^ and 512 cm^−1^ bands are associated with the coupling of the tetragonal phase-dependent TO modes, corresponding to the [*A*_1_(TO)] and [*A*_1_(TO), *E*(TO)] phonon modes, respectively [37,38]. The peak at 715 cm^−1^ represents the [*A*_1_(LO), *E*(LO)] phonon mode, which belongs to the coupling between the LO modes with the highest frequency of *A*_1_ symmetry [39,40]. The relation curve between the area of the normalized peak at 305 cm^−1^ band representing the characteristic peak of the tetragonal phase and *c/a* is shown in Figure 2b. The satisfactory linear fitting results between the area of the normalized peak and *c/a* further confirm the local tetragonal characteristics of the BT sample, which is consistent with the macroscopic structure obtained by XRD.

**Figure 3:**
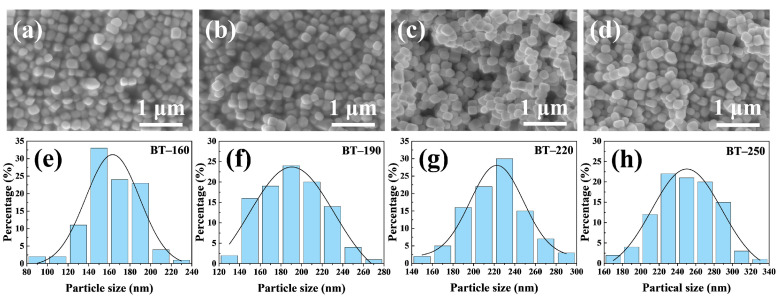
SEM images of (**a**) BT–160, (**b**) BT–190, (**c**) BT–220, and (**d**) BT–250 samples. Corresponding particle size distribution histogram of (**e**) BT–160, (**f**) BT–190, (**g**) BT–220, and (**h**) BT–250 samples.

The morphology and homogeneity of BTPs were evaluated by SEM images containing a mass of particles. The SEM images in Figure 3a–d show that four BT powders exhibit rounded cube morphology and show good uniformity. In addition to the tetragonal properties, ethanol solution also has a positive effect on the dispersion of BTPs. In general, the narrow size distribution and dispersion of particles are obtained by the hydrophobic interaction between the long hydrophobic chains of surfactant molecules, leading to the anchoring of surfactant chains on the surface of the sample [41]. However, BTPs with good dispersion were obtained by regulating the hydrothermal process without using surfactants/stabilizing agents in this work. The presence of ethanol in hydrothermal media could decrease the polarity of the solution to promote the formation of BTPs. In addition, the use of ethanol could also reduce the particle interfacial activity after the formation of BTPs to prevent the aggregation and abnormal growth of particles to ensure their good dispersion and homogeneity [42]. Furthermore, the formation of nearly monodisperse and uniform BTPs is also attributed to the uniform nucleation of continuous heating at higher temperatures [43,44]. As shown in Figure S10 and Table S3, the absolute values of the Zeta potential of BT samples are greater than 30 mV, which proves that the dispersion is good [45,46]. Good uniformity reduces the statistical error of the average particle size of samples and benefits the performance of the assembled MLCCs. Slight agglomeration occurs in the BT–250 sample while the overall dispersibility is still good (Figure 3d), which may be due to the boiling and volatilization of ethanol during the hydrothermal reaction.

To quantify the uniformity of BTPs, the particle sizes of 100 particles in the SEM image were measured to ensure the reliability of the results. The principle is to measure and record the sum of the particle sizes of 100 particles and divide by a number of particles to obtain the average value. The histograms of the particle size distribution of each sample are shown in Figure 3e–g, which are located below the corresponding SEM images. The fitting curves of the particle size distribution histograms in Figure 3e–g show that the particle size distributions of four synthesized BT samples obey the Gaussian distribution. From the size distributions, it can be clearly concluded that the average particle sizes of four samples gradually increase with the extension of the hydrothermal time. The average particle size of the BT–160 sample with the shortest hydrothermal duration is about 160 nm, while the particle size is mainly centralized around 250 nm when the hydrothermal time reaches 50 h.

**Figure 4:**
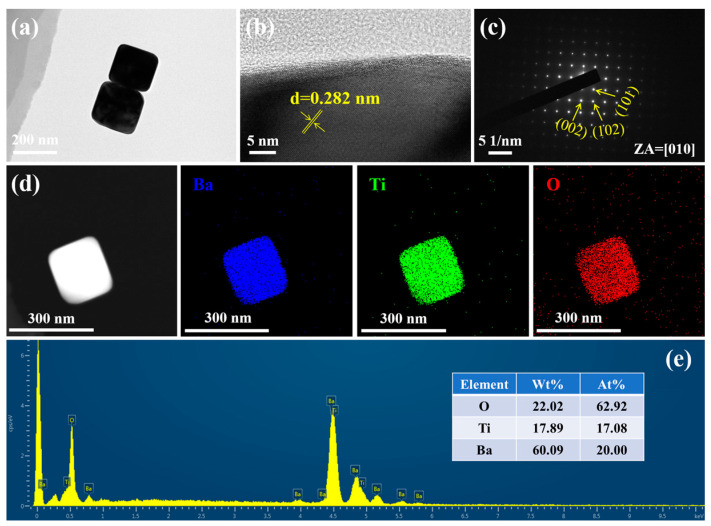
(**a**) TEM image; (**b**) HRTEM image; (**c**) SAED pattern; (**d**) scanning transmission electron microscope (STEM) image and EDS element mapping analysis results; (**e**) EDS results of BT–190 sample.

The microstructure and composition of BTPs were further characterized by TEM and element mapping analysis, as shown in Figure 4. Taking the BT–190 sample as an example, Figure 4a shows that the particles are in the shape of cubes with rounded corners, and the particle size is consistent with the results obtained from the SEM image. The clear lattice fringes in the HRTEM image (Figure 4b) demonstrate the good crystallinity of the BT–190 sample. Through Fast Fourier transform (FFT) and inverse FFT, the HRTEM image of Figure 4b is converted into a measurable lattice fringe of 0.282 nm, which is ascribed to the (110) crystal plane of tetragonal BT. A single crystal structure can be revealed from the SAED pattern in which the diffraction spots with marks are indexed as (002), (102), and (101) crystal planes of tetragonal BT when the zone axis (ZA) is in the [010] direction. Meanwhile, the clear single-crystal diffraction pattern also verifies its excellent crystallinity [47]. Uniform element distribution and homogeneous particle sizes are important prerequisites for the reliability of MLCCs. The EDS mapping analysis of STEM images in Figure 4d and Figure S6–S8 show that Ba, Ti, and O elements are evenly distributed in the particle. Furthermore, the EDS spectra and elemental quantitative result of the BT–190 sample are shown in Figure 4e. The atomic percentage of O (62.92%), Ti (17.08%), and Ba (20.00%) elements is about 3:1:1, which is consistent with the atomic ratio of BT (BaTiO_3_) [48]. In Figure S9, the EDS spectra and elemental quantification results of BT–160, BT–220, and BT–250 samples show similar 3:1:1 atomic percentages of O, Ti, and Ba elements.

**Figure 5:**
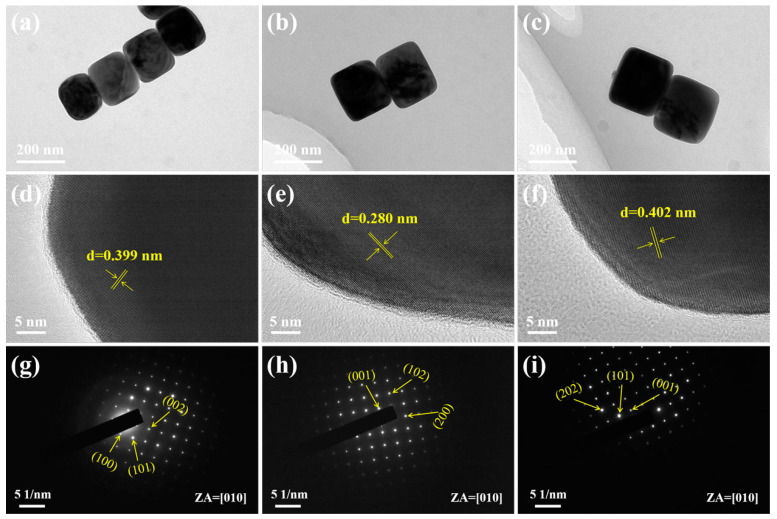
(**a**–**c**) TEM images; (**d**–**f**) HRTEM images and (**g**–**i**) SAED patterns of BT–160, BT–220, and BT–250 samples.

TEM, HRTEM, and SAED characterization for BT–160, BT–220, and BT–250 samples were compared to demonstrate the microscopic crystal structures and crystallinity. In the TEM images of BT–160, BT–220, and BT–250 samples in Figure 5a–c, the particle sizes of the circular cubes are consistent with the corresponding SEM particle size statistics. The average particle size of BTPs increases with the increase in hydrothermal time, but the shape characteristic of rounded corners gradually weakens to the shape of cubes closer. These are more evident in TEM images. BTPs show smooth surfaces in SEM and TEM, and no pores are observed. The clear lattice fringes of HRTEM images in Figure 5d–f and SAED patterns in Figure 5g–i indicate that all BT samples show good crystallinity. The measured lattice fringe spacings in Figure 5d–f of random particles from BT–160, BT–220, and BT–250 samples are 0.399, 0.280, and 0.402 nm, corresponding to (100), (110), and (001) crystal planes of tetragonal BT in turn. The SAED patterns in Figure 5g–i demonstrate that the BT samples present a single crystal structure, and the clear diffraction spots are also indexed to the tetragonal structure. TEM analysis results are not only consistent with XRD and Raman results in tetragonal phase analysis but also consistent with SEM results in particle size and morphology analysis. The results show that the BT–160, BT–190, BT–220, and BT–250 samples synthesized by a facile and eco-friendly hydrothermal method achieve the balance of high tetragonal, controllable particle size and uniform morphology. It is still worth mentioning that the regulation of particle size and high tetragonality of BT powders in this work is more effective than most of the reported hydrothermal preparation results in recent literature (Table S4). In Figure S9, about 28 g BT products show the possibility of easy large-scale preparation. Additionally, the raw materials do not require strict storage conditions, which improves the practical application potential to a higher degree.

**Figure 6:**
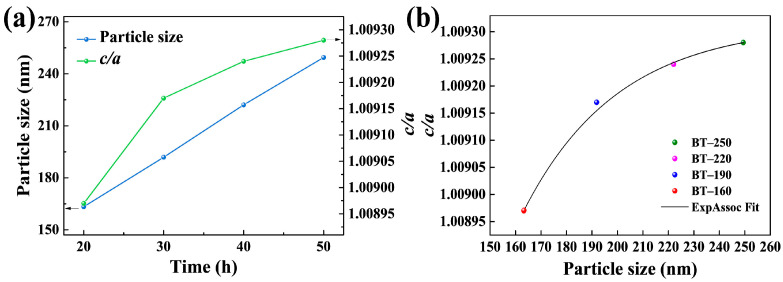
(**a**) The curves of particle size and *c/a* with hydrothermal time, respectively; (**b**) The fitting curve of particle size with *c/a*.

Furthermore, the variation trends among hydrothermal parameters, particle size, and tetragonality were discussed. The hydrothermal temperature is set to 260 °C. On the one hand, it contributes to the formation of the tetragonal phase. On the other hand, a slightly higher temperature reduces the internal defects of hydrothermal BTPs and facilitates the exploration of the particle size dependence of their tetragonality [29]. The particle size and tetragonality of BT samples in Figure 6a increase with hydrothermal time, but the trend is slightly different. The tetragonality increases slowly with hydrothermal time, indicating that the effect of hydrothermal time on tetragonality is limited. However, the role of ethanol ensures that all samples retain high tetragonality. The trend of the average particle size and tetragonality is presented in Figure 6b, which also reflects the stability of the experiment.

**Figure 7:**
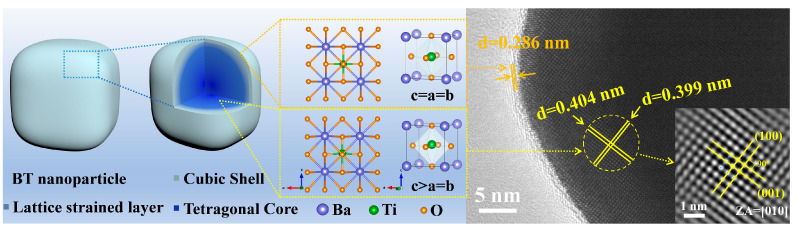
Schematic diagram of the core–shell structure of the synthesized BTPs. The inset is an inverse FFT atomic reconstruction.

In order to investigate the root cause of the trend of average particle size and tetragonality, the internal structure of BTPs was further studied. The presence of the surface effect makes BTPs exhibit a core–shell structure (Figure 7) consisting of the tetragonal core (the blue region in the center), the transition lattice strain layer (the bluish-grey region in the middle), and the surface cubic shell (the grey region in the outer layer), which is based on the Ginsburg–Landau–Devonshire thermodynamic theory study [49]. The shell is formed due to structural distortions caused by surface relaxation, and the weakening of the surface effect leads to the properties of the diffusive phase transition inside BTPs [49,50]. Similarly, the size-dependent expansion is caused by the tetragonal BT, while the thermal expansion coefficient of the cubic phase is almost unchanged, which is also because the cubic phase only exists on the surface of the particles [27].

The lattice spacings of the shell and core are measured via HRTEM to verify the difference between the internal and surface phase structures. The results show that the lattice spacings of 0.399 nm for the core and 0.286 nm for the shell correspond to the (100) crystal plane of tetragonal phase BT (PDF#05-0626) and the (110) crystal plane of cubic phase BT (PDF#31-0174), respectively. This result is consistent with the theoretical explanation of the core–shell structure of BTPs. The atomic arrangement of BTPs is reconstructed by inverse FFT processing, and the crystal structure diagrams of cubic and tetragonal BT are presented accordingly. The eccentric displacement of Ti atoms is observed in the tetragonal BT structure and leads to the change of lattice parameters. For BT crystals with cubic morphology, the volume fraction of the interior tetragonal core is provided with the relation (1):

(1) $$\mathrm{core}\%=100\times {\left(1-\frac{2L}{D}\right)}^{3}$$

where *D* is the particle size of BTPs and *L* is the cubic shell thickness [50]. Since smaller BTPs have higher specific surface energy, which is more favorable for the formation of cubic shells [27]. Additionally, the variation of *D* is much larger than that of *L*. Therefore, from this relation, the volume fraction of the tetragonal nuclei increases with the particle size. The trend of tetragonality and particle size in Figure 6b is consistent with this relation, confirming the dependence of tetragonality on particle size.

## 4. Conclusions

In summary, four BT powders with high tetragonality, controllable particle size, and uniform morphology are successfully prepared by a facile and eco-friendly hydrothermal method under the optimal solvent condition of water: ethanol: ammonia solution of 2:2:1. The analysis of crystal structure and tetragonal characteristic indicates that the tetragonality of all BT samples is about 1.009 and increases with the hydrothermal time. The results of morphology and particle size show that BT powders of 160, 190, 220, and 250 nm exhibit good homogeneity and dispersion. The presence of ethanol not only affects the hydroxylation process to ensure high tetragonality but also reduces the interfacial activity of BTPs to maintain good uniformity and dispersion of BTPs. The core–shell structure is revealed by the different lattice fringe spacings of the core and edge of BTPs and the crystal structure given by the reconstructed atomic arrangement via inverse FFT processing, which further confirms the size dependence of tetragonality. These results provide new insights into the regulation of BT properties and eco-friendly synthesis processes, which will facilitate wider practical applications.

## Data Availability

Data are contained within the article.

## Supplementary Materials

The following supporting information can be downloaded at: https://www.mdpi.com/article/10.3390/ma16114191/s1, Figure S1–S5: XRD patterns of BT powders; Figure S6–S8: STEM images and EDS elements mapping of BT powders; Figure S9: EDS results of BT powders; Figure S10: Zeta potential curves of BT powders. Figure S11: Photographs of BT powders; Table S1: Specific values of the lattice parameters *a*, *c*, and their *c/a* ratios for BT powders; Table S2: The ratios between tetragonal and cubic phases of BT powders; Table S3: Zeta potentials of BT powders. Table S4: Comparison of dual regulations of tetragonality and particle size prepared by the hydrothermal method. References [51,52,53,54,55] are cited in the Supplementary Materials.

## References

[1] Song E., Kim D.H., Jeong E.J., Choi M., Kim Y., Jung H.J., Choi M.Y. (2021). Effects of Particle Size and Polymorph Type of TiO_2_ on the Properties of BaTiO_3_ Nanopowder Prepared by Solid-State Reaction. Environ. Res..

[2] Qi H., Fang L., Xie W., Zhou H., Wang Y., Peng C. (2015). Study on the Hydrothermal Synthesis of Barium Titanate Nano-Powders and Calcination Parameters. J. Mater. Sci. Mater. Electron..

[3] Zhu C., Cai Z., Guo L., Li L., Wang X. (2020). Grain Size Engineered High-Performance Nanograined BaTiO_3_-Based Ceramics: Experimental and Numerical Prediction. J. Am. Ceram. Soc..

[4] Zhang X., Yue J., Zhao Y., Yan Z., Zhu G., Liu L., Xu H., Yu A. (2021). Synthesis of Tetragonal BaTiO_3_ Nano-Particle Via a Novel Tartaric Acid Co-Precipitation Process. Ceram. Int..

[5] Usher T.-M., Kavey B., Caruntu G., Page K. (2020). Effect of BaCO_3_ Impurities on the Structure of BaTiO_3_ Nanocrystals: Implications for Multilayer Ceramic Capacitors. ACS Appl. Nano Mater..

[6] Gromada M., Biglar M., Trzepieciński T., Stachowicz F. (2017). Characterization of BaTiO_3_ Piezoelectric Perovskite Material for Multilayer Actuators. Bull. Mat. Sci..

[7] On D.V., Vuong L.D., Chuong T.V., Quang D.A., Tung V.T. (2021). Study on the Synthesis and Application of BaTiO_3_ Nanospheres. Int. J. Mater. Res..

[8] Zhang W., Xu H., Zhu G., Yu A. (2015). Synthesis and Characterization of Tetragonal Barium Titanate Powders by an Ion-Exchange and Hydrothermal Process. J. Ceram. Process. Res..

[9] Navas D., Fuentes S., Castro-Alvarez A., Chavez-Angel E. (2021). Review on Sol-Gel Synthesis of Perovskite and Oxide Nanomaterials. Gels.

[10] Cui X.C., Wang J., Zhang X.Y., Wang Q., Song M.M., Chai J.L. (2019). Preparation of Nano-TiO_2_ by a Surfactant-Free Microemulsion–Hydrothermal Method and Its Photocatalytic Activity. Langmuir.

[11] Özen M., Mertens M., Snijkers F., Cool P. (2016). Hydrothermal Synthesis and Formation Mechanism of Tetragonal Barium Titanate in a Highly Concentrated Alkaline Solution. Ceram. Int..

[12] Ding S.S., Chen L., Liao J., Huo Q., Wang Q., Tian G., Yin W.Y. (2023). Harnessing Hafnium-Based Nanomaterials for Cancer Diagnosis and Therapy. Small.

[13] Hao Y., Feng Z., Banerjee S., Wang X., Billinge S.J.L., Wang J., Jin K., Bi K., Li L. (2021). Ferroelectric State and Polarization Switching Behaviour of Ultrafine BaTiO_3_ Nanoparticles with Large-Scale Size Uniformity. J. Mater. Chem. C.

[14] Su C.Y., Otsuka Y., Huang C.Y., Hennings D.F., Pithan C., Shiao F.T., Waser R. (2013). Grain Growth and Crystallinity of Ultrafine Barium Titanate Particles Prepared by Various Routes. Ceram. Int..

[15] Li R.J., Wei W.X., Hai J.L., Gao L.X., Gao Z.W., Fan Y.Y. (2013). Preparation and Electric-Field Response of Novel Tetragonal Barium Titanate. J. Alloy. Compd..

[16] Lee J., Jeong H., Ma S. (2022). Effects of Annealing Temperature on Structural Phase Transition and Microstructure Evolution of Hydrothermally Synthesized Barium Titanate Nanoparticles. Mater. Res. Express.

[17] Zhang W., Feng Q., Hosono E., Asakura D., Miyawaki J., Harada Y. (2020). Tetragonal Distortion of a BaTiO_3_/Bi_0.5_Na_0.5_TiO_3_ Nanocomposite Responsible for Anomalous Piezoelectric and Ferroelectric Behaviors. ACS Omega.

[18] Wang Y., Wen X., Jia Y., Huang M., Wang F., Zhang X., Bai Y., Yuan G., Wang Y. (2020). Piezo-Catalysis for Nondestructive Tooth Whitening. Nat. Commun..

[19] Fang C., Zhou D., Gong S., Luo W. (2010). Multishell Structure and Size Effect of Barium Titanate Nanoceramics Induced by Grain Surface Effects. Phys. Status Solidi B.

[20] Nakashima K., Onagi K., Kobayashi Y., Ishigaki T., Ishikawa Y., Yoneda Y., Yin S., Kakihana M., Sekino T. (2021). Stabilization of Size-Controlled BaTiO_3_ Nanocubes Via Precise Solvothermal Crystal Growth and Their Anomalous Surface Compositional Reconstruction. ACS Omega.

[21] Peng Y., Chen H., Shi F., Wang J. (2020). Effect of Polyethylene Glycol on BaTiO_3_ Nanoparticles Prepared by Hydrothermal Preparation. IET Nanodielectr..

[22] Maček Kržmanc M., Bračko I., Budič B., Suvorov D., Parans Paranthaman M. (2013). The Morphology Control of BaTiO_3_ Particles Synthesized in Water and a Water/Ethanol Solvent. J. Am. Ceram. Soc..

[23] Habib A., Stelzer N., Angerer P., Haubner R. (2011). Effect of Temperature and Time on Solvothermal Synthesis of Tetragonal BaTiO_3_. Bull. Mat. Sci..

[24] Pang X.X., Wang T.T., Liu B., Fan X.Y., Liu X.R., Shen J., Zhong C., Hu W.B. (2023). Effect of Solvents on the Morphology and Structure of Barium Titanate Synthesized by a One-Step Hydrothermal Method. Int. J. Miner., Metall. Mater..

[25] Pfaff G. (1991). BaTiO_3_ Preparation by Reaction of TiO_2_ with Ba(OH)_2_. J. Eur. Ceram. Soc..

[26] Jain S., Khire V.H., Kandasubramanian B. (2019). Barium Titanate: A Novel Perovskite Oxide Burning Rate Modifier for Htpb/Ap/Al Based Composite Propellant Formulations. Propellants Explos. Pyrotech..

[27] Han M., Rong Y., Li Q., Xing X., Kang L. (2015). Thermal Expansion of Nano-Sized BaTiO_3_. CrystEngComm.

[28] Baek C., Yun J.H., Wang H.S., Wang J.E., Park H., Park K.-I., Kim D.K. (2018). Enhanced Output Performance of a Lead-Free Nanocomposite Generator Using BaTiO_3_ Nanoparticles and Nanowires Filler. Appl. Surf. Sci..

[29] Hayashi H., Ebina T. (2018). Effect of Hydrothermal Temperature on the Tetragonality of BaTiO_3_ Nanoparticles and in-Situ Raman Spectroscopy under Tetragonal Cubic Transformation. J. Ceram. Soc. Jpn..

[30] Baek C., Wang J.E., Moon S., Choi C.-H., Kim D.K., Riman R. (2016). Formation and Accumulation of Intragranular Pores in the Hydrothermally Synthesized Barium Titanate Nanoparticles. J. Am. Ceram. Soc..

[31] Noma T., Wada S., Yano M., Suzuki T. (1996). Analysis of Lattice Vibration in Fine Particles of Barium Titanate Single Crystal Including the Lattice Hydroxyl Group. J. Appl. Phys..

[32] Ji X., Zhu Y., Lian X., Fan B., Liu X., Xiao P., Zhang Y. (2022). Hydroxylation Mechanism of Phase Regulation of Nanocrystal BaTiO_3_ Synthesized by a Hydrothermal Method. Ceram. Int..

[33] Venkateswaran U.D., Naik V.M., Naik R. (1998). High-Pressure Raman Studies of Polycrystalline BaTiO_3_. Phys. Rev. B.

[34] DiDomenico M., Wemple S.H., Porto S.P.S., Bauman R.P. (1968). Raman Spectrum of Single-Domain BaTiO_3_. Phys. Rev..

[35] Shi T., Xie L., Gu L., Zhu J. (2015). Why Sn Doping Significantly Enhances the Dielectric Properties of Ba(Ti_1−X_ Sn_x_)O_3_. Sci. Rep..

[36] Xu L., Zhu K., Wang J., Gu Q., Cao Y., Zheng H., Liu J., Qiu J. (2015). Microwave-Assisted Sol–Hydrothermal Synthesis of Tetragonal Barium Titanate Nanoparticles with Hollow Morphologies. J. Mater. Sci. Mater. Electron..

[37] Wang L., Lv J., Shi F., Song K., Lei W., Zhou H., Qi Z.-M., Wang J. (2021). Intrinsic Dielectric Properties and Lattice Vibrational Characteristics of Single Phase BaTiO_3_ Ceramic. J. Mater. Sci. Mater. Electron..

[38] Küçük Ö., Teber S., Cihan Kaya İ., Akyıldız H., Kalem V. (2018). Photocatalytic Activity and Dielectric Properties of Hydrothermally Derived Tetragonal BaTiO_3_ Nanoparticles Using TiO_2_ Nanofibers. J. Alloy. Compd..

[39] Amaechi I.C., Katoch R., Kolhatkar G., Sun S., Ruediger A. (2020). Particle Size Effect on the Photocatalytic Kinetics of Barium Titanate Powders. Catal. Sci. Technol..

[40] Cao Y., Zhu K., Liu J., Qiu J. (2014). Fabrication of BaTiO_3_ Nanoparticles and Its Formation Mechanism Using the High Temperature Mixing Method under Hydrothermal Conditions. Adv. Powder Technol..

[41] Sonkusare V.N., Chaudhary R.G., Bhusari G.S., Mondal A., Potbhare A.K., Mishra R.K., Juneja H.D., Abdala A.A. (2020). Mesoporous Octahedron-Shaped Tricobalt Tetroxide Nanoparticles for Photocatalytic Degradation of Toxic Dyes. ACS Omega.

[42] Yan Y., Xia H., Fu Y., Xu Z., Ni Q.-Q. (2020). Controlled Hydrothermal Synthesis of Different Sizes of BaTiO_3_ Nano-Particles for Microwave Absorption. Mater. Res. Express.

[43] Potbhare A.K., Chaudhary R.G., Chouke P.B., Yerpude S., Mondal A., Sonkusare V.N., Rai A.R., Juneja H.D. (2019). Phytosynthesis of Nearly Monodisperse Cuo Nanospheres Using Phyllanthus Reticulatus/Conyza Bonariensis and Its Antioxidant/Antibacterial Assays. Mater. Sci. Eng. C.

[44] Chouke P.B., Potbhare A.K., Meshram N.P., Rai M.M., Dadure K.M., Chaudhary K., Rai A.R., Desimone M.F., Chaudhary R.G., Masram D.T. (2022). Bioinspired Nio Nanospheres: Exploring in Vitro Toxicity Using Bm-17 and L. Rohita Liver Cells, DNA Degradation, Docking, and Proposed Vacuolization Mechanism. ACS Omega.

[45] Taheri M., Zanca B., Dolgos M., Bryant S., Trudel S. (2021). Water-Dispersible and Ferroelectric Pegylated Barium Titanate Nanoparticles. Mater. Adv..

[46] Yaseen H., Baltianski S., Tsur Y. (2006). Effect of Incorporating Method of Niobium on the Properties of Doped Barium Titanate Ceramics. J. Am. Ceram. Soc..

[47] Sheng Y.Q., Li W.L., Xu L.L., Zhu Y.F. (2022). High Photocatalytic Oxygen Evolution Via Strong Built-in Electric Field Induced by High Crystallinity of Perylene Imide Supramolecule. Adv. Mater..

[48] Choi I., Lee S.J., Kim J.C., Kim Y.G., Hyeon D.Y., Hong K.S., Suh J., Shin D., Jeong H.Y., Park K.I. (2020). Piezoelectricity of Picosecond Laser-Synthesized Perovskite BaTiO_3_ Nanoparticles. Appl. Surf. Sci..

[49] Fang C., Zhou D., Gong S. (2011). Core-Shell Structure and Size Effect in Barium Titanate Nanoparticle. Phys. B.

[50] Pasuk I., Neatu F., Neatu S., Florea M., Istrate C.M., Pintilie I., Pintilie L. (2021). Structural Details of BaTiO_3_ Nano-Powders Deduced from the Anisotropic XRD Peak Broadening. Nanomaterials.

[51] Zamperlin N., Ceccato R., Fontana M., Pegoretti A., Chiappini A., Dire S. (2021). Effect of Hydrothermal Treatment and Doping on the Microstructural Features of Sol-Gel Derived BaTiO_3_ Nanoparticles. Materials.

[52] Meng H., Chen Z., Lu Z., Wang X., Fu X. (2021). Hydrothermal Synthesis of Tetragonal Barium Titanate Nanopowders under Moderate Conditions. Process. Appl. Ceram..

[53] Chen H., Wang J., Yin X., Xing C., Li J., Qiao H., Shi F. (2019). Hydrothermal Synthesis of BaTiO_3_ Nanoparticles and Role of Pva Concentration in Preparation. Mater. Res. Express.

[54] Li J., Inukai K., Tsuruta A., Takahashi Y., Shin W. (2018). Synthesis of Highly Disperse Tetragonal BaTiO_3_ Nanoparticles with Core-Shell by a Hydrothermal Method. J. Asian. Ceram. Soc..

[55] Huang Y.A., Lu B., Li D.D., Tang Z.H., Yao Y.B., Tao T., Liang B., Lu S.G. (2017). Control of Tetragonality Via Dehydroxylation of BaTiO_3_ Ultrafine Powders. Ceram. Int..

