# Supplementary material for: A Facile and Eco-Friendly Hydrothermal Synthesis of High Tetragonal Barium Titanate with Uniform and Controllable Particle Size
Source: Materials (Basel). 2023 Jun 5;16(11):4191. doi: 10.3390/ma16114191 (PMC10254630; doi:10.3390/ma16114191)
Supplement: Supplementary file 1 [file materials-16-04191-s001.zip › materials-2391885-supplementary.pdf]

Supplementary Materials

A facile and eco-friendly hydrothermal synthesis of high tetragonal barium titanate with uniform and controllable particle size

Tingting Wang [1], Xiaoxiao Pang [1], Bin Liu [1], Jie Liu [1], Jing Shen [2] and Cheng Zhong [1,3,*]

[1] Key Laboratory of Advanced Ceramics and Machining Technology (Ministry of Education), School of Materials Science and Engineering, Tianjin University, Tianjin 300072, China; wtt@tju.edu.cn (T.W.); xiaoxiao_pang17@163.com (X.P.); arthurlou@tju.edu.cn (B.L.); jieliu0109@tju.edu.cn (J.L.).

[2] Chongqing Newcent New Materials Co., Ltd. Chongqing 401147, China; shenjing@chinanewcent.com (J.S.).

[3] Joint School of National University of Singapore and Tianjin University, International Campus of Tianjin University, Binhai New City, Fuzhou 350207, China.

* Correspondence: cheng.zhong@tju.edu.cn

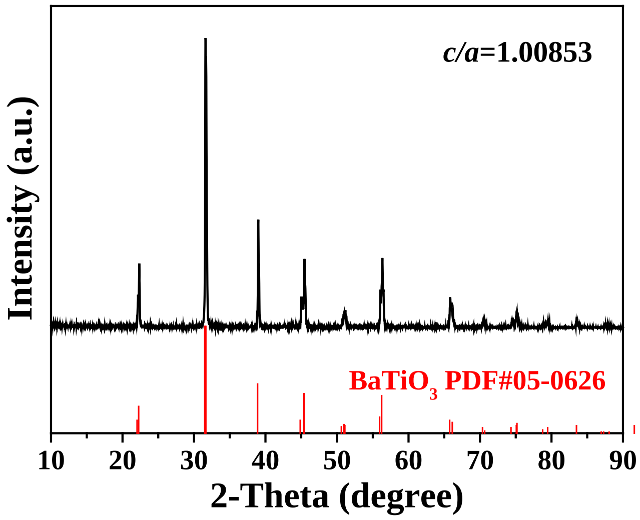

Figure S1. XRD pattern of BT powders. The hydrothermal solvent consists of 30 mL of water, 10 mL of ethanol and 10 mL of ammonia solution (3 : 1 : 1).

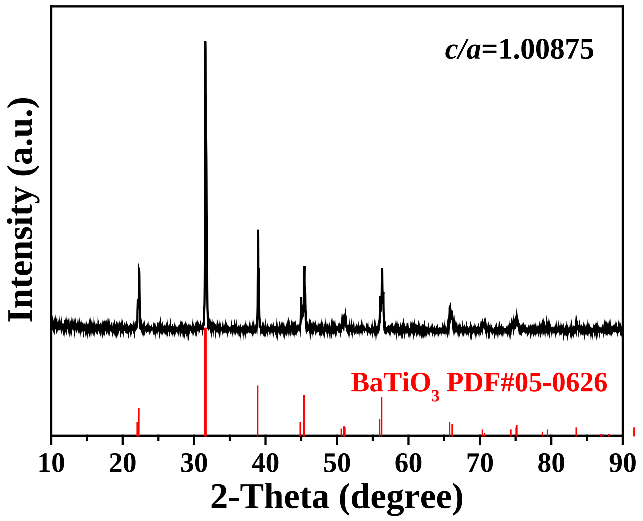

Figure S2. XRD pattern of BT powders. The hydrothermal solvent consists of 25 mL of water, 15 mL of ethanol and 10 mL of ammonia solution (5 : 3 : 2).

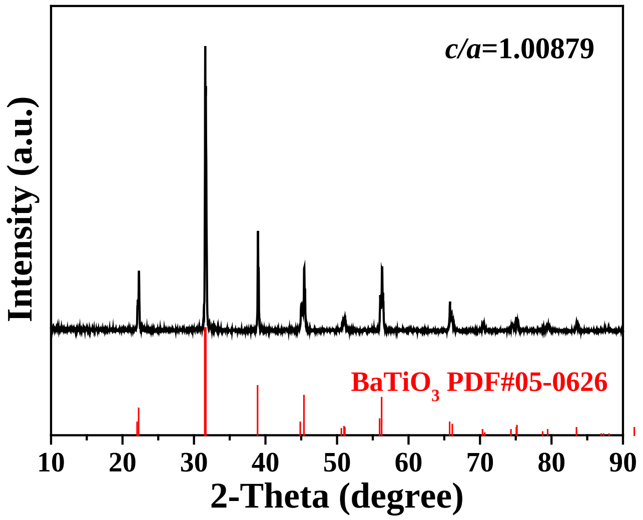

Figure S3. XRD pattern of BT powders. The hydrothermal solvent consists of 15 mL of water, 25 mL of ethanol and 10 mL of ammonia solution (3 : 5 : 2).

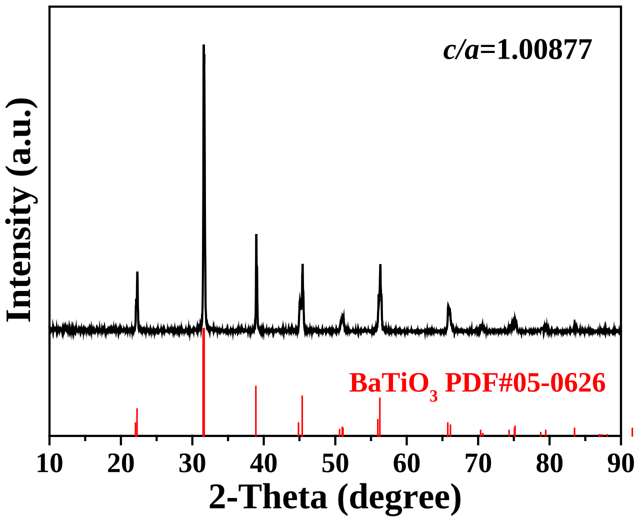

Figure S4. XRD pattern of BT powders. The hydrothermal solvent consists of 10 mL of water, 30 mL of ethanol and 10 mL of ammonia solution (1 : 3 : 1).

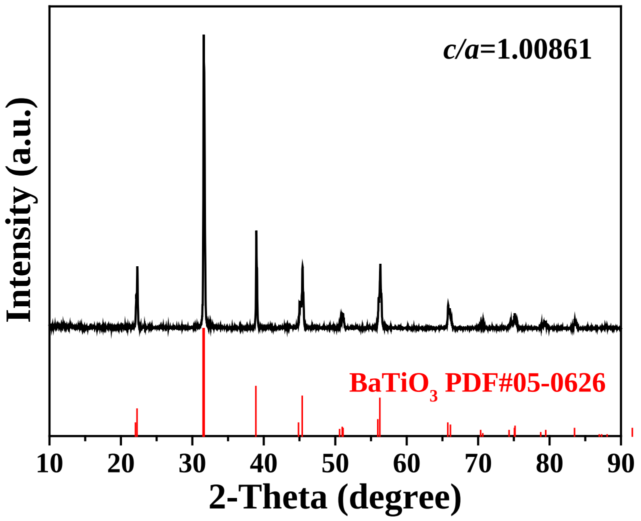

Figure S5. XRD pattern of BT powders. The hydrothermal solvent consists of 5 mL of water, 35 mL of ethanol and 10 mL of ammonia solution (1 : 7 : 2).

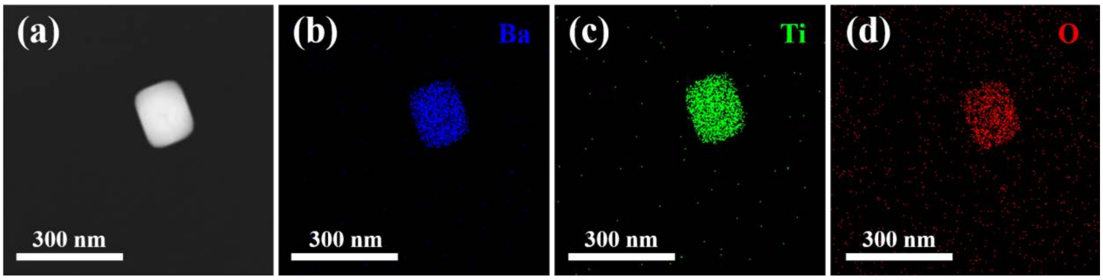

Figure S6. (**a**) STEM image and (**b–d**) EDS elements mapping of BT–160 sample.

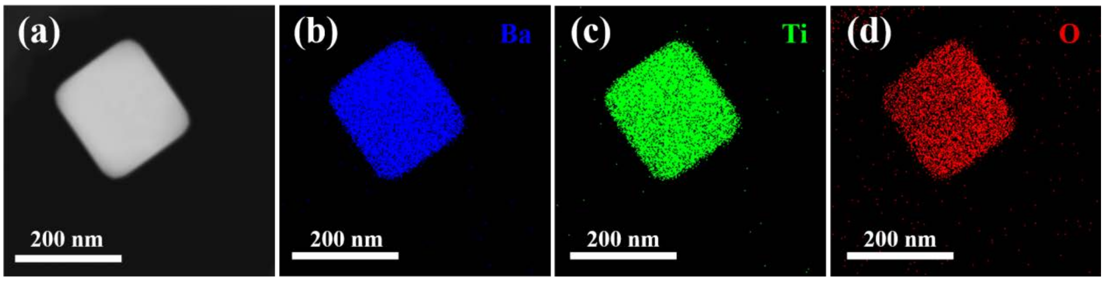

Figure S7. (**a**) STEM image and (**b–d**) EDS elements mapping of BT–220 sample.

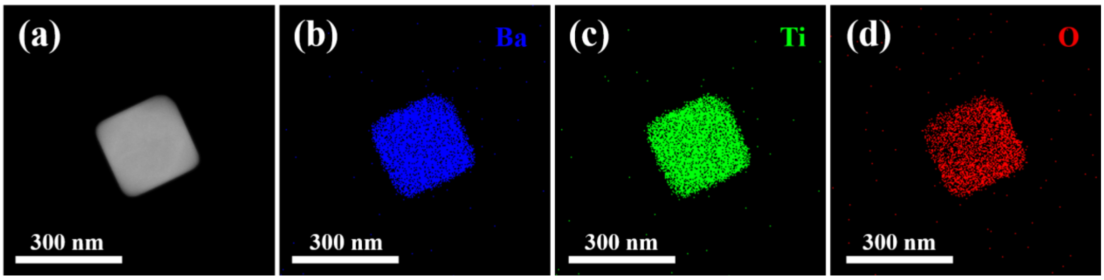

Figure S8. (**a**) STEM image and (**b–d**) EDS elements mapping of BT–250 sample.

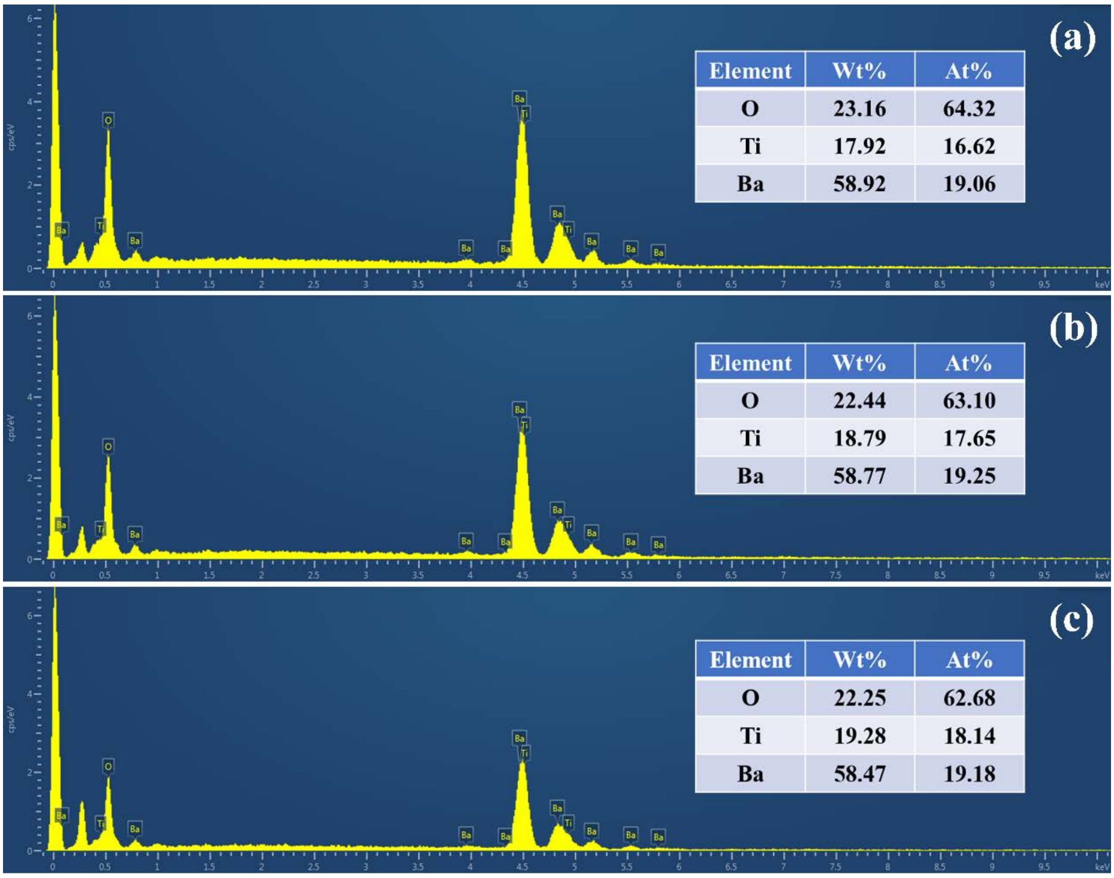

(a)

Element	Wt%	At%
O	23.16	64.32
Ti	17.92	16.62
Ba	58.92	19.06

(b)

Element	Wt%	At%
O	22.44	63.10
Ti	18.79	17.65
Ba	58.77	19.25

(c)

Element	Wt%	At%
O	22.25	62.68
Ti	19.28	18.14
Ba	58.47	19.18

Figure S9. EDS results of (**a**) BT–160, (**b**) BT–220 and (**c**) BT–250 samples.

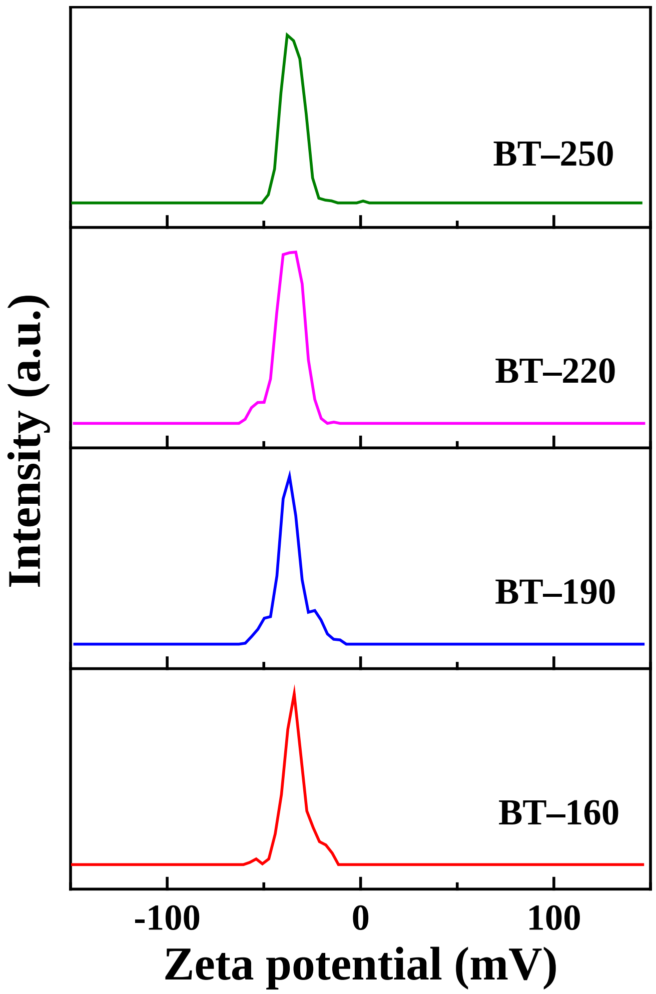

Figure S10. Zeta potential curves of four BT powders.

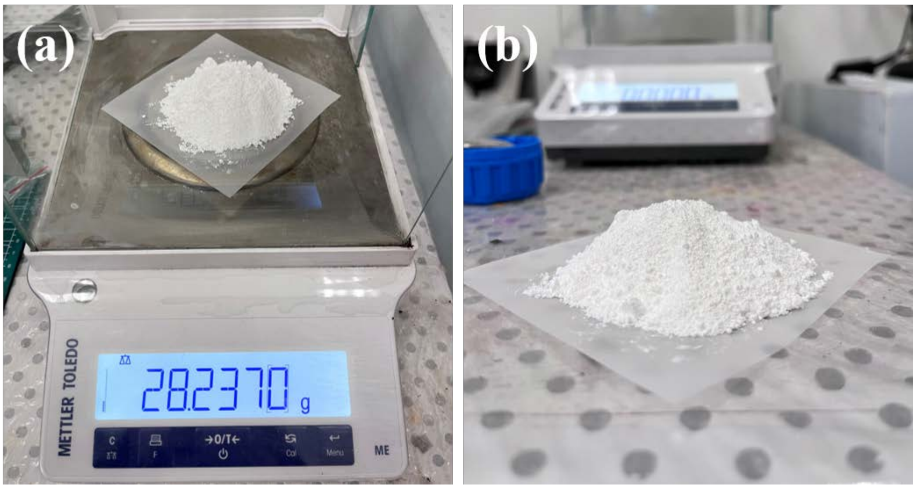

Figure S11. Photographs of (**a**) about 28 g of BT–190 product and (**b**) BT powders.

Table S1 Specific values of the lattice parameters *a, c* and their *c*/*a* ratios for four BT powders.

Samples	BT–160	BT–190	BT–220	BT–250
a	3.99292	3.99430	3.99400	3.99317
c	4.02874	4.03093	4.03091	4.03024
c/*a*	1.00897	1.00917	1.00924	1.00928

Table S2 The ratios between tetragonal and cubic phases of four BT powders.

Samples	BT–160	BT–190	BT–220	BT–250
Tetragonal phase	89.8%	91.3%	92.3%	92.9%
Cubic phase	10.2%	8.7%	7.7%	7.1%

Table S3. Zeta potentials of four BT powders.

Samples	BT–160	BT–190	BT–220	BT–250
Zeta potential (mV)	-33.6	-36.1	-37.0	-34.9

Table S4 Comparison of dual regulations of tetragonality and particle size prepared by the hydrothermal method.

Raw materials	*c*/*a*	particle size (nm)	Ref.
TiO_2 and $Ba(OH)_2{\cdot}8H_2O$	1.00897–1.00924	160–250	This work
TiO_2 and $BaCl_2$	1.001–1.007	21–512	[1]
$Ti(O^iPr)_4$ and $Ba(CH_3COO)_2$	1.0063–1.0075	117–162	[2]
$Ti(OC_4H_9)_4$ and $Ba(OH)_2{\cdot}H_2O$	1.0028–1.0073	96.1	[3]
TiO_2 and $Ba(OH)_2{\cdot}8H_2O$	1.000–1.007	69.8–119.0	[4]
$(Ti(OC_4H_9)_4$ and $Ba(OH)_2{\cdot}8H_2O$	–	57–75	[5]
TiO_2 and $Ba(OH)_2{\cdot}8H_2O$	1.006–1.008	90–120	[6]
$TiCl_4$ and $BaCl_2{\cdot}2H_2O$	1.0001–1.0061	47–83	[7]
$Ti[OC_4H_9]_4$ and $Ba(CH_3COO)_2$	1.0056–1.0076	85.18–112.12	[8]

References

1 Yan, Y.; Xia, H.; Fu, Y.; Xu, Z.; Ni, Q.-Q. Controlled Hydrothermal Synthesis of Different Sizes of $BaTiO_3$ Nano-Particles for Microwave Absorption. *Mater. Res. Express* **2020**, *6*, 1250i3. https://doi.org/10.1088/2053-1591/ab6daf

2 Zamperlin, N.; Ceccato, R.; Fontana, M.; Pegoretti, A.; Chiappini, A.; Dire, S. Effect of Hydrothermal Treatment and Doping on the Microstructural Features of Sol-Gel Derived $BaTiO_3$

Nanoparticles. *Materials* **2021**, *14*, 4345. https://doi.org/10.3390/ma14154345

3 Meng, H.; Chen, Z.; Lu, Z.; Wang, X.; Fu, X. Hydrothermal Synthesis of Tetragonal Barium Titanate Nanopowders under Moderate Conditions. *Process. Appl. Ceram.* **2021**, *15*, 179–183. https://doi.org/10.2298/pac2102179m

4 Hayashi, H.; Ebina, T. Effect of Hydrothermal Temperature on the Tetragonality of $BaTiO_3$ Nanoparticles and in-Situ Raman Spectroscopy under Tetragonal-Cubic Transformation. *J. Ceram. Soc. Jpn.* **2018**, *126*, 214–220. https://doi.org/10.2109/jcersj2.17125

5 Chen, H.; Wang, J.; Yin, X.; Xing, C.; Li, J.; Qiao, H.; Shi, F. Hydrothermal Synthesis of $BaTiO_3$ Nanoparticles and Role of Pva Concentration in Preparation. *Mater. Res. Express* **2019**, *6*, 055028. https://doi.org/10.1088/2053-1591/ab0520

6 Habib, A.; Stelzer, N.; Angerer, P.; Haubner, R. Effect of Temperature and Time on Solvothermal Synthesis of Tetragonal $BaTiO_3$. *Bull. Mater. Sci.* **2011**, *34*, 19–23. https://doi.org/10.1007/s12034-011-0043-2

7 Li, J.; Inukai, K.; Tsuruta, A.; Takahashi, Y.; Shin, W. Synthesis of Highly Disperse Tetragonal $BaTiO_3$ Nanoparticles with Core-Shell by a Hydrothermal Method. *J. Asian. Ceram. Soc.* **2018**, *5*, 444–451. https://doi.org/10.1016/j.jascer.2017.09.006

8 Huang, Y.A.; Lu, B.; Li, D.D.; Tang, Z.H.; Yao, Y.B.; Tao, T.; Liang, B.; Lu, S.G. Control of Tetragonality Via Dehydroxylation of $BaTiO_3$ Ultrafine Powders. *Ceram. Int.* **2017**, *43*, 16462–16466. https://doi.org/10.1016/j.ceramint.2017.09.027
